# Determinants of binge eating and its impact on indicators of obesity among Finnish adolescents - a cohort study

**DOI:** 10.1186/s40337-024-01181-y

**Published:** 2024-12-23

**Authors:** Reetta Lehto, Monica Ålgars, Sohvi Lommi, Marja H Leppänen, Heli Viljakainen

**Affiliations:** 1 Folkhälsan Research Center, Helsinki, Finland; 2 Department of Food and Nutrition, University of Helsinki, Helsinki, Finland; 3 Department of Psychology, University of Helsinki, Helsinki, Finland; 4 Faculty of Medicine, University of Helsinki, Helsinki, Finland; 5 Institute of Biomedicine, School of Medicine, University of Eastern Finland, Kuopio, Finland

**Keywords:** Youth, Children, Binge eating, Disordered eating, Family, Risk factors, Determinants, Weight

## Abstract

**Background:**

Binge eating, a type of disordered eating, is relatively common among youth and predisposes them to many adverse health outcomes. Diverse psychological and social factors may be associated with binge eating. The aim of this study was to examine child and parental psychosocial determinants of binge eating and its impact on indicators of obesity.

**Methods:**

The sample consisted of 10 679 Finnish adolescents who participated in the Fin-HIT cohort study. The participants were 9–12 years at baseline, and they were followed-up for 2.6 years on average. At baseline, children’s anthropometry was measured, and they reported binge eating, disordered eating attitudes and behaviors, self-esteem, and body shape satisfaction with validated questionnaires. Parents reported their own weight satisfaction, dieting, and depressive symptoms at baseline. Adjusted logistic and linear regressions were used in the analyses.

**Results:**

Self-esteem was associated with lower odds (OR 0.88, 95% CI 0.85–0.91), and overall disordered eating attitudes and behaviors were associated with higher odds (OR 1.08, 95% CI 1.06–1.10) of binge eating. Additionally, child and parent body dissatisfaction were associated with binge eating but not in the fully adjusted model where the child’s BMI was additionally considered. None of the parental factors were associated with binge eating in the fully adjusted model. Binge eating was associated with higher BMI z-scores and waist circumferences both cross-sectionally and longitudinally.

**Conclusions:**

In support of previous findings, we found that low self-esteem, body dissatisfaction and other disordered eating attitudes and behaviors are associated with binge eating, but our results also highlight the impact of weight status as a potential moderator when the determinants of binge eating are examined. More studies on the role of parental factors in adolescent binge eating are needed.

## Abstract (plain language summary)

Binge eating is a type of disordered eating characterized by uncontrollable eating episodes that often negatively affects mental and physical health. We investigated the individual- and parent-related factors linked to binge eating and whether binge eating is related to BMI and waist circumference among adolescents. We studied 10,696 Finnish adolescents who were 9–12 years old at the beginning of the study. The participants’ weight, height and waist circumference were measured, and they and their parents completed a questionnaire. The participants were followed up for 2.6 years on average. We found that low self-esteem and general disordered eating attitudes and behaviors were linked to binge eating. Additionally, body dissatisfaction present in the child or the parent was associated with the child’s binge eating, but this depended on the child’s BMI. Binge eating was repeatedly associated with a higher BMI and waist circumference.

## Introduction

Disordered eating is prevalent among children and adolescents worldwide, as more than one-fifth of children and adolescents display some symptoms of disordered eating [1]. This poses a notable risk for developing an eating disorder, and subsequent mental and physical adversities, such as mood and anxiety disorders, substance use disorders, self-harm, obesity, and type 2 diabetes, accompanied with it [2–4]. Binge eating (BE) is a form of disordered eating that seems to have increased in prevalence in recent years [5]. BE refers to episodes of objectively eating large amounts of food in a discrete period of time combined with a feeling of loss of control over eating [6]. In clinical eating disorders, BE is a shared symptom of binge eating disorder and bulimia nervosa [7].

In recent years, BE has been divided into subcategories to reflect different features of behavior. Subjective BE refers to eating episodes with a feeling of loss of control over eating without eating objectively large amounts of food [8], whereas objective BE refers to BE that includes a combination of objectively large amounts of food and a feeling of loss of control. In addition, several recent studies have examined loss of control (LOC) eating, which refers only to loss of control without assessing the amount of food eaten [9–11]. The benefit of examining LOC eating, especially among children and adolescents, is that it may be easier to measure in epidemiological studies since evaluating an objectively large amount of food among growing children is challenging, and LOC eating may be as relevant for eating disorder pathology, psychopathology, and impaired functioning as objective BE [8, 9, 11, 12]. In this study, objective BE is examined and referred to as BE, but in previous studies, all forms of BE and LOC eating are referred to.

The prevalence of BE, which is usually self-reported in epidemiological studies, has varied from 2% to approximately 20% among US and European children and adolescents, with older adolescents having a higher prevalence [2, 12–15]. Binge and LOC eating are associated with several adverse health outcomes among children and adolescents: subsequent binge eating disorder, worsening of eating disorder pathology, obesity, cardiometabolic risk factors irrespective of weight, and mental health problems [3, 16–18]. However, at least half of children and adolescents with binge or LOC eating may remit without intervention [2, 3].

The determinants of adolescent binge or LOC eating are various, including physiological, social, emotional, and environmental factors [11, 19]. Children’s psychological and psychosocial determinants, e.g., low self-esteem, body dissatisfaction, depressive symptoms, negative affect, and dieting, have been reported to be associated with binge or LOC eating in cross-sectional and longitudinal studies [2, 9, 10, 19, 20]. BE is also usually associated with overall disordered eating attitudes and behaviors (DEAB) [8–10].

In addition to child characteristics, many family factors, e.g., parental characteristics or interpersonal relationships in the family, may act as determinants of binge or LOC eating. A systematic review of 15 studies on children under 12 years of age revealed that consistent familial determinants for childhood BE were weight-related teasing and parental emotional unresponsiveness [21]. Inconsistent results have been reported regarding, e.g., parents’ disordered eating, body dissatisfaction, and maternal dieting [21–23]. The role of parental mental health problems in association with children’s BE or other disordered eating is not yet fully understood. A few studies have shown a possible association: a Swedish register-based study revealed that a diagnosed mental health disorder among parents was associated with a diagnosed eating disorder in their 12–24-year-old children [24], and another study revealed that higher parental depression and anxiety were associated with greater DEAB among adolescents who had poorer emotion regulation skills [25]. However, null findings also exist [12].

Although relatively many studies exist on the correlates of binge and LOC eating among youth, very few studies have examined determinants of BE among Finnish or other Nordic adolescents, accounting for possibly different cultural contexts. Such cultural differences could be e.g. children often spending afternoons without adult company in Finland, as school days are quite short and usually both parents work full-time. This could enable development of disordered eating behaviors. Additionally, more knowledge on parental factors in relation to child BE is needed. The objectives of this study are first to examine the psychosocial and parental determinants of BE among Finnish youth in the age group of 9–12 years and second to examine its associations with indicators of obesity cross-sectionally and longitudinally with a 2.5-year follow-up.

## Materials and methods

### Sample and data collection

This study uses data from the Finnish Health in Teens (Fin-HIT) cohort study as a secondary analysis. Fin-HIT examines adolescents’ health behaviors, wellbeing, and weight in a prospective design [26]. The study was approved by the Ethics Committee of the Hospital District of Helsinki and Uusimaa (decision number 169/13/03/00/10). Written informed consent was obtained from all the participating children and their guardians. The data collection procedure is detailed elsewhere [26]. At baseline, in 2011–2014, a total of 11 407 children participated in the study, which were 30% of all children contacted. Recruitment was conducted, and data were collected mainly from schools. In addition, a parent of 6046 children (53% of the child participants) completed a parental questionnaire at baseline. A follow-up was conducted in 2015–2016 with 5911 participants (54% of the baseline participants). The follow-up data collection was conducted with home-sent questionnaires. The children were 9–12 years old at baseline, and the mean follow-up time was 2.6 years (SD 0.8). The analytic sample of this study consists of children who answered the BE questions in the child questionnaire at baseline (*n* = 10 679, 94% of all baseline participants).

### Study outcomes and exposures

**Binge eating** was assessed at baseline with two questions in the child’s questionnaire: (1) During the past year, how often have you eaten so much food in a short period of time that you would be embarrassed if others saw you? The answer options were “Never” (1); “A couple of times” (2); “Less than once a month” (3); “1–3 times a month” (4); “Once a week” (5); and “More than once a week” (6). If the child chose answer options 2–6, they were advised to answer the following consecutive question: (2) Did you feel out of control, like you couldn’t stop eating even if you wanted to stop? The answer options were “yes” or “no”. The participants were categorized as adolescents with BE if they reported having BE episodes at least once a month (selecting answer options 3–6 in question 1 and yes to question 2). The questionnaire has been validated among 9–14-year-olds in the US [27].

**Body dissatisfaction** was assessed with a pictorial tool, which has been described in detail elsewhere [28]. The children were asked which of the seven sex-specific pictures illustrating different body sizes most resembled their current body shape and which of the pictures most resembled the body shape they wanted [29]. Based on their answers, the children were categorized as those satisfied with their body shape (their body shape was equal to the body shape they wanted), those wanting to be thinner, and those wanting to be larger than they were. Those wanting to be thinner and those wanting to be larger were compared separately against those who were satisfied with their body shape.

**Self-esteem** was assessed with the widely used Rosenberg Self-Esteem Scale [30]. The scale includes 10 statements with four response options ranging from 1 (strongly disagree) to 4 (strongly agree). The scale was used as a continuous variable ranging from 10 to 40 points, with higher points reflecting better self-esteem. Owing to slight modifications in the questionnaire during the baseline data collection, the self-esteem measure was not available for the whole sample (missing *n* = 1231, 12% of the analytic sample). The self-esteem scale indicated good internal consistency, with a Cronbach’s alpha of 0.81.

Other **disordered eating attitudes and behaviors (DEAB)** were assessed with a 26-item Children’s Eating Attitude Test (ChEAT) [31] containing statements on eating behavior and attitudes toward foods, eating, and losing weight, with six answer options ranging from always to never, with scores ranging from 0 to 3. The ChEAT has previously been validated among Finnish children of similar age, and a 24-item version of the questionnaire was found to be more internally consistent than the original 26-item questionnaire [32]. Since one of the items in the remaining 24-item version specifically concerning LOC eating was also assessed in the BE questions, the item was omitted (I have eaten plenty of food, and I have felt that I might not be able to stop eating). Thus, a 23-item version, after omitting items 4, 19, and 25 of the original ChEAT, was used. The Cronbach’s alpha for the 23 questions was 0.84, indicating good internal consistency. In the analyses, the sum score of the 23 items of the ChEAT was used as a continuous variable (range 0–69), with higher scores reflecting more frequent DEAB.

**Parental depression** was assessed with a modified Finnish version of the Beck Depression Inventory [33, 34] containing 13 items with 5 answer options (R-BDI) [35]. The validity and reliability of the R-BDI are good [36]. The scores of each item (0–3) were summed (range 0–39), and higher scores indicate more depressive symptoms. The Cronbach’s alpha for the items was 0.77, indicating good internal consistency.

**Parental weight dissatisfaction** was assessed with one question: I think I… (1) am about the right weight; (2) should gain weight; and (3) should lose weight. The answers were categorized as “satisfied with weight” (answer option 1), which was used as a reference group, and “wants to lose weight” (answer option 3). Those wanting to gain weight (*n* = 66, 1% of the parents) were excluded from the analyses concerning parental weight dissatisfaction.

**Parental dieting** was assessed with a question: During the past year, did you try to lose weight? The answer options were “yes” or “no”.

**Body mass index (BMI) z score and waist circumference.** In the main baseline sample, the children’s weight, height, and waist circumference were measured at school by trained field workers. In the pilot sample and during the follow-up, parents were instructed to measure and report children’s weight, height, and waist circumference with a home-sent measuring tape. Self-reported weight and height in this age group have been found to be valid [37]. International Obesity Task Force (IOTF)-based BMI z scores (BMIz) were calculated and used in the analyses [38].

### Covariates

The child’s age and sex were used as confounding factors. Age and sex were confirmed by linking with the National Population Information System at the Population Register Center.

### Statistical analyses

T tests and chi-square tests were used to assess differences between the groups with and without BE. With a cross-sectional design, we conducted logistic regression analyses with BE as the outcome and body dissatisfaction, self-esteem and DEAB (child determinants), and with parental depression, weight dissatisfaction and dieting (parent determinants) as independent variables. Three models were used: 1) each independent variable separately in the model while adjusting for age and sex; 2a) age, sex, and all child-level independent variables in the same model; 2b) age, sex, and all parent-level independent variables in the same model; and 3) additionally adjusting for BMIz. Associations are indicated with odds ratios (ORs) and 95% confidence intervals (CIs). Analyses of child-level and parental determinants were conducted separately because of the significantly smaller sample size in the parental data (child data *n* = 10 679 vs. parental data *n* = 6046). The associations of BE with BMIz and waist circumference were examined with linear regression both cross-sectionally and longitudinally with 2 models: (1) crude and (2) adjusted for age and sex. In the longitudinal analyses, the duration of follow-up and baseline BMIz and waist circumference were also adjusted for. In addition, in the analyses concerning waist circumference, height was also adjusted for. Associations are indicated with b coefficients and 95% CIs. Statistical analyses were conducted with IBM SPSS Statistics version 29.0. A significance level of 0.05 was used. Pairwise deletion was used for missing values.

## Results

### Sample characteristics

At baseline, the mean age of the participants was 11.2 (SD 0.9) years, the age range was 8.7–13.9 years, and 52.6% of the participants were girls. Mean duration of follow-up was 2.6 years. In total, 1.8% (*n* = 193) of the participants reported BE at least once a month (referred to as adolescents with BE). At baseline, participants with BE had lower self-esteem, more DEAB, higher BMIz and waist circumference and were more likely to be dissatisfied with their body shape than adolescents without BE (Table 1). Additionally, at follow-up, adolescents with BE had higher BMIz values and waist circumferences than did those without BE. Among the parental variables, weight dissatisfaction was more common among parents of adolescents with BE than among those without BE (66% vs. 51%).

**Table 1:** Description of the studied variables among the participants with and without binge eating

	Binge eating		No binge eating		
	*n* = 193		*n* = 10 503		
**CHILD**	Mean (SD)/%	missing	Mean (SD)/%	missing	P*
**Baseline**					
Age	11.0 (1.0)	1	11.2 (0.8)	16	0.002
Gender					0.12
girls	58.0	0	52.4	0	
boys	42.0	0	47.6	0	
Waist circumference, cm	69.4 (11.2)	2	64.1 (7.9)	57	< 0.001
BMI z-score	0.88 (1.13)	2	0.21 (1.00)	105	< 0.001
Self-esteem (10–40)	25.6 (5.1)	11	30.0 (4.6)	1234	< 0.001
DEAB^1^ (0–69)	8.9 (8.6)	0	2.4 (4.2)	0	< 0.001
Body dissatisfaction		1		42	< 0.01
satisfied with body shape	34.9%		61.1%		
wants to be thinner	58.9%		29.4%		
wants to be larger	6.3%		9.4%		
**Follow-up**					
Duration of follow-up, years	2.4 (0.6)	116	2.6 (0.8)	5153	0.003
Waist circumference, cm	75.4 (11.1)	119	70.7 (8.1)	5228	< 0.001
BMI z-score	0.95 (0.99)	117	0.29 (0.92)	5220	0.001
**PARENT**					
**Baseline**					
Depressive symptoms (0–39)	2.1 (3.6)	117	1.6 (2.8)	4931	0.15
Dieting		117		4912	0.15
Yes	61.8%		53.5%		
No	38.2%		46.5%		
Weight dissatisfaction		118		4974	0.01
satisfied with weight	34.2%		49.4%		
wants to lose weight	65.8%		50.6%		

*for difference between adolescents with and without binge eating. T-test or Chi-square test. ^1^ Disordered eating attitudes and behaviors

.

### Cross-sectional associations of child and parental characteristics with child BE

**Fig. 1:**
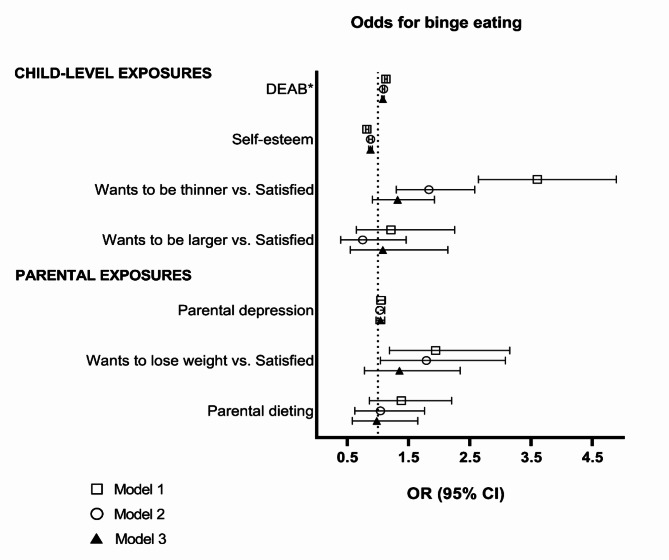
Associations of child and parental determinants and binge eating. Logistic regression, odds ratios and 95% confidence intervals. *DEAB = disordered eating attitudes and behaviors. Model 1 adjusted for age and sex. Model 2 adjusted for age, sex, and all other independent variables (separately for child-level variables and parental variables). Model 3 adjusted for model 2 variables + BMI z score

Figure 1 shows the cross-sectional associations of child and parental determinants with BE. In all the models, higher self-esteem was associated with lower odds (Model 3 (fully adjusted model): OR 0.88, 95% CI 0.85–0.91), and other DEABs associated with higher odds (Model 3: OR 1.08, 95% CI 1.06–1.10) for BE. Compared with being satisfied with their body shape, wanting to be thinner was associated with higher odds for BE in models 1 and 2, but the association was no longer statistically significant after adjusting for BMIz (Model 3). Parental weight dissatisfaction was associated with higher odds for child BE in models 1 and 2 (Model 2 OR: 1.79, 95% CI 1.04–3.08), but the association disappeared when child BMIz was added in Model 3.

### Cross-sectional and longitudinal associations of BE with indicators of obesity

Table 2 shows the cross-sectional and longitudinal associations of BE with indicators of obesity. BE was associated with higher BMIz (Model 2: b 0.67, 95% CI 0.53–0.82) and waist circumference (Model 2: b 5.43, 95% CI 4.47–6.39) cross-sectionally. In addition, BE predicted higher BMIz (Model 2: b 0.14, 95% CI 0.02–0.26) and waist circumference (Model 2: b 1.11, 95% CI 0.00–2.22) after the 2.6-year follow-up period when adjusting for the baseline BMIz or waist circumference, respectively.

**Table 2:** Linear regression analyses predicting BMI z-score and waist circumference at baseline and at follow-up by baseline binge eating

	Baseline										Follow-up									
	Model 1					Model 2					Model 1					Model 2				
	BMI z-score										BMI z-score									
	b	t	p	R^2^	n	b	t	p	R^2^	n	b	t	p	R^2^	n	b	t	p	R^2^	n
**Binge eating**	0.67	9.22	< 0.001	0.01	10,589	0.67	9.28	< 0.001	0.01	10,589	0.14	2.27	0.02	0.69	5255	0.14	2.31	0.02	0.69	5230
	**Waist circumference**										**Waist circumference**									
**Binge eating**	5.33	10.85	< 0.001	0.24	10,610	5.43	11.08	< 0.001	0.27	10,593	0.98	1.72	0.09	0.65	5312	1.11	1.96	0.05	0.65	5284

Baseline BMI z-score Model 1 = no adjustments, Model 2 = adjusted for age and sex. Follow-up BMI z-score Model 1 = adjusted for baseline BMI z-score, Model 2 = adjusted for baseline BMI z-score, age, sex, and duration of follow-up. Baseline Waist circumference Model 1 = adjusted for height, Model 2 = adjusted for height, age, and sex. Follow-up Waist circumference Model 1 = adjusted for follow-up height and baseline waist circumference. Model 2 = adjusted for follow-up height, baseline waist circumference, age, sex, and duration of follow-up

## Discussion

This study examined whether child and parental psychosocial factors were cross-sectionally associated with BE and whether BE was associated with BMIz and waist circumference cross-sectionally and longitudinally among young Finnish adolescents. The prevalence of BE was 1.8% at baseline. Low self-esteem and other disordered eating attitudes and behaviors were associated with greater odds for child BE when adjusting for the child’s BMIz. Child and parental body dissatisfaction were associated with higher odds of BE, but not after adjusting for child BMIz. As expected, BE was associated with higher BMIz and waist circumference cross-sectionally and longitudinally after a 2.6-year follow-up, although the association was diluted at follow-up.

Similarly to our results, low self-esteem has been found to be associated with and to predict binge and LOC eating in several studies among children and adolescents [2, 9, 14]. In previous studies, low self-esteem has also been associated with body dissatisfaction and, e.g., depression, and these factors together may mediate the effects of one another on BE. For example, self-esteem and depression have been found to mediate associations between body dissatisfaction and BE [14, 39, 40]. Additionally, according to research findings, physical appearance is a component of general self-esteem [41]. Thus, it is noteworthy that in our analyses, contrary to Brechan et al. (2015), the associations between self-esteem and body dissatisfaction also remained significant when analyzed together, showing independent associations between them and BE. As expected, other DEABs were also associated with higher odds of BE. Previous studies have also revealed that different disordered eating symptoms often coexist and may contribute to each other, e.g., dietary restraint and BE [8–10, 12].

We found that wanting to be thinner was associated with higher odds of BE, but the association was attenuated after controlling for BMIz. Wanting to be larger was not associated with BE. Many previous studies, both cross-sectional and longitudinal, have reported similar associations, but mostly no adjustments with weight status have been made [2, 9, 12, 14, 39, 42]. Studies exploring body image have mostly used statements that have referred to wanting to be thinner, or no distinction between wanting to be thinner or larger has been made [2, 9, 12, 14]. The disappearance of the association after adjustment for BMIz suggests that BMIz explained the association between body dissatisfaction and BE. Body dissatisfaction as a determinant of BE may be especially pronounced among adolescents with higher weight, but other determinants may be more important for normal weight adolescents with BE. Thus, stratified analyses based on weight status may be warranted.

In summary, the current results suggest that low self-esteem, body dissatisfaction, and overall disordered eating attitudes and behaviors play important roles in adolescent BE. Binge eating is a multifaceted behavior influenced by various factors, including psychological, social, biological, and environmental aspects. Therefore, holistic approach is needed when assessing risks or developing preventive interventions for binge eating. Low self-esteem, overall disordered eating attitudes, and body dissatisfaction may be important driving forces, but they cannot be seen as the only ones. With respect to prevention, body image and self-esteem have been targeted in interventions aiming to prevent eating disorders among children and adolescents with some success [43–45].

The associations between binge and LOC eating and higher BMI have been reported in many studies [2, 10, 12, 18], including ours. In addition, among youth with obesity, LOC eating is a common feature, as one quarter of children and adolescents with overweight and obesity demonstrate LOC eating [46]. This association may also be bidirectional, as overweight can be a consequence of BE as well as a factor contributing to it [2, 12, 18], e.g., due to dieting attempts [47] or weight bullying/negative comments predisposing individuals to BE [48]. The genetic predisposition to overweight may also play a role, as it has been found to be associated with BE [49]. In this study, BE was strongly associated with higher BMI and waist circumference cross-sectionally, and it also predicted higher BMI and waist circumference, although the impact was diluted, predicting only 0.14 units higher BMIz and 1.1 cm larger waist circumference after follow-up. This may be due to possible changes in BE or other disordered eating behaviors during the follow-up, which we were unfortunately unable to consider due to a lack of data.

We found little evidence that potential parental correlates are associated with child BE, as neither parental depressive symptoms nor dieting were associated with child BE in the current study. Similarly, maternal or paternal depressive symptoms were not associated with child LOC or BE in a Dutch study of 14-year-olds, although the authors state that these results should be interpreted with caution because of the use of a possibly selective sample [12]. Martinson et al. (2016) reported that parental depression and anxiety were associated with adolescent DEAB, but only among adolescents with poorer emotional awareness/expression [25]. The prevalence of having any eating disorder was elevated among 12–24-year-old offspring of parents with a diagnosed mental illness [24]. Thus, the role of parental depression in child BE needs further clarification.

In this study, parental weight dissatisfaction was associated with higher odds for child BE, but the association was attenuated after controlling for child BMIz. Parental and child BMIs as well as parental and child body dissatisfaction are probably intertwined and may together predispose the child to BE. In this study, children of parents with weight dissatisfaction had higher BMIz values and wanted to be thinner more likely than children of parents who were satisfied with their weight (data not shown). Previous results have shown that such indirect encouragement to lose weight, such as maternal/parental body dissatisfaction, weight concerns, and dieting, is deleterious for a child’s body image and eating behaviors and attitudes, especially among daughters [22, 50–52]. Our results partly support these findings but also stress the importance of the child’s and perhaps also the parent’s weight status in this matter.

The prevalence of BE in the present study, 1.8%, was lower than that reported in other studies on adolescents. In other community sample studies, the prevalence has varied between 2.2% and over 20% among US, German, Dutch, and Italian adolescents [2, 12–15]. The low prevalence might be due to the young study population, as the prevalence of BE usually peaks in late adolescence [13], or somewhat different assessment methods. We found no other Finnish or Nordic studies for comparison.

The findings of the study, together with previous studies, highlight the need for preventive measures in families, schools and among health care practitioners to support good self-esteem, positive body image, and healthy eating behaviors and attitudes among adolescents. In addition, scalable preventive interventions targeting these determinants are also needed.

### Strengths and limitations

A strength of the study is the large and geographically diverse sample, although the sample is not representative of all Finnish 9–12-year-olds owing to the higher socioeconomic background of the families compared with the general population [53]. Another strength is the use of validated questions and questionnaires concerning BE, DEAB, and self-esteem [27, 32, 54]. The study also adds to the previous literature, as there are very few studies on child/adolescent BE and its determinants in Finland and other Nordic countries.

Although validated, the use of the two-item assessment method to assess BE can also be seen as a limitation. Using structured interviews would be the best option to assess BE among youth, as they may have difficulty identifying BE episodes when they do not receive detailed instructions [55]. This limitation concerns most questionnaire-based studies, as BE is usually estimated with two questions: one on the amount of food eaten and the other on the feeling of loss of control. Another limitation is the descriptive nature of most of the analyses. There is a possibility for inverse causality concerning the child’s psychosocial determinants of BE and its cross-sectional associations with obesity indicators, although the results of other studies shed light on the direction of the associations.

## Conclusions

Among Finnish adolescents, having low self-esteem and experiencing other disordered eating attitudes and behaviors was associated with an increased likelihood of BE, irrespective of adolescent weight. In addition, child and parental body dissatisfaction were associated with child BE but not independently of child weight. Thus, weight status may act as a moderator in the associations between possible determinants and BE. Our findings support previous studies on the topic in Europe, but this is among the first studies on the topic in Nordic countries. Given the many negative implications of BE for child and youth health and well-being, efforts should be put in its prevention in schools, primary health care, and families by targeting its known modifiable determinants. In addition, continued research efforts to identify yet unknown risk factors and to prevent and treat BE are needed.

## Data Availability

The datasets used and analyzed during the current study are available from the corresponding author upon reasonable request.

## Abbreviations

BE: Binge eating
BMI: Body mass index
BMIz: Body mass index z score
CI: Confidence interval
DEAB: Disordered eating attitudes and behaviors
LOC: Loss-of-control
OR: Odds ratio

## References

[1] López-Gil JF, García-Hermoso A, Smith L, Firth J, Trott M, Mesas AE, et al. Global Proportion of Disordered Eating in Children and Adolescents: A Systematic Review and Meta-analysis. JAMA Pediatr. 2023;177(4):363–72. 36806880 10.1001/jamapediatrics.2022.5848 PMC9941974

[2] Goldschmidt AB, Wall MM, Zhang J, Loth KA, Neumark-Sztainer D. Overeating and binge eating in emerging adulthood: 10-year stability and risk factors. Dev Psychol. 2016;52(3):475–83. 26689758 10.1037/dev0000086 PMC4760881

[3] Hilbert A, Brauhardt A. Childhood loss of control eating over five-year follow-up. Int J Eat Disord. 2014;47(7):758–61. 24899359 10.1002/eat.22312

[4] Keski-Rahkonen A, Mustelin L. Epidemiology of eating disorders in Europe: prevalence, incidence, comorbidity, course, consequences, and risk factors. Curr Opin Psychiatry. 2016;29(6):340–5. 27662598 10.1097/YCO.0000000000000278

[5] Mitchison D, Touyz S, González-Chica DA, Stocks N, Hay P. How abnormal is binge eating? 18-Year time trends in population prevalence and burden. Acta Psychiatr Scand. 2017;136(2):147–55. 28419425 10.1111/acps.12735

[6] Kjeldbjerg ML, Clausen L. Prevalence of binge-eating disorder among children and adolescents: a systematic review and meta-analysis. Eur Child Adolesc Psychiatry. 2021. 10.1007/s00787-021-01850-2 34318368

[7] American Psychiaric Association. Diagnostic and statistical manual of mental disorders. 5th ed. Arlington, VA, 2013.

[8] Brownstone LM, Bardone-Cone AM. Subjective binge eating: a marker of disordered eating and broader psychological distress. Eat Weight Disord. 2021;26(7):2201–9. 33200355 10.1007/s40519-020-01053-9

[9] Goossens L, Soenens B, Braet C. Prevalence and Characteristics of Binge Eating in an Adolescent Community Sample. J Clin Child Adolesc Psychol. 2009;38(3):342–53. 19437295 10.1080/15374410902851697

[10] Schlüter N, Schmidt R, Kittel R, Tetzlaff A, Hilbert A. Loss of control eating in adolescents from the community. Int J Eat Disord. 2016;49(4):413–20. 26711325 10.1002/eat.22488

[11] Byrne ME, LeMay-Russell S, Tanofsky-Kraff M. Loss-of-Control Eating and Obesity Among Children and Adolescents. Curr Obes Rep. 2019;8(1):33–42. 30701372 10.1007/s13679-019-0327-1

[12] Derks IPM, Harris HA, Staats S, Gaillard R, Dieleman GC, Llewellyn CH, et al. Subclinical binge eating symptoms in early adolescence and its preceding and concurrent factors: a population-based study. J Eat Disord. 2022;10(1):180. 36424658 10.1186/s40337-022-00688-6 PMC9685858

[13] Skinner HH, Haines J, Austin SB, Field AE. A prospective study of overeating, binge eating, and depressive symptoms among adolescent and young adult women. J Adolesc Health. 2012;50(5):478–83. 22525111 10.1016/j.jadohealth.2011.10.002 PMC3336086

[14] Sehm M, Warschburger P. Prospective Associations Between Binge Eating and Psychological Risk Factors in Adolescence. J Clin Child Adolesc Psychol. 2018;47(5):770–84. 27399285 10.1080/15374416.2016.1178124

[15] Laghi F, Bianchi D, Pompili S, Lonigro A, Baiocco R. Binge eating and binge drinking behaviors: the role of family functioning. Psychol Health Med. 2021;26(4):408–20. 32228049 10.1080/13548506.2020.1742926

[16] Tanofsky-Kraff M, Shomaker LB, Olsen C, Roza CA, Wolkoff LE, Columbo KM, et al. A prospective study of pediatric loss of control eating and psychological outcomes. J Abnorm Psychol. 2011;120(1):108–18. 21114355 10.1037/a0021406 PMC3051193

[17] Radin RM, Tanofsky-Kraff M, Shomaker LB, Kelly NR, Pickworth CK, Shank LM, et al. Metabolic characteristics of youth with loss of control eating. Eat Behav. 2015;19:86–9. 26210388 10.1016/j.eatbeh.2015.07.002 PMC4644474

[18] Sonneville KR, Horton NJ, Micali N, Crosby RD, Swanson SA, Solmi F, et al. Longitudinal Associations Between Binge Eating and Overeating and Adverse Outcomes Among Adolescents and Young Adults: Does Loss of Control Matter? JAMA Pediatr. 2013;167(2):149. 23229786 10.1001/2013.jamapediatrics.12 PMC3654655

[19] Simone M, Scodes J, Mason T, Loth K, Wall MM, Neumark-Sztainer D. Shared and non-shared risk and protective factors of binge eating and binge drinking from adolescence to young adulthood. J Health Psychol. 2021;26(6):805–17. 31014132 10.1177/1359105319844588 PMC6813845

[20] Stice E, Gau JM, Rohde P, Shaw H. Risk factors that predict future onset of each DSM-5 eating disorder: Predictive specificity in high-risk adolescent females. J Abnorm Psychol. 2017;126(1):38–51. 27709979 10.1037/abn0000219 PMC5215960

[21] Saltzman JA, Liechty JM. Family correlates of childhood binge eating: A systematic review. Eat Behav. 2016;22:62–71. 27089384 10.1016/j.eatbeh.2016.03.027

[22] Laboe AA, Hocking JE, Gondoli DM. Body dissatisfaction and disordered eating within the mother-daughter dyad: An actor-partner interdependence approach. Body Image. 2022;43:25–33. 35994997 10.1016/j.bodyim.2022.08.004

[23] Hartmann AS, Czaja J, Rief W, Hilbert A. Psychosocial risk factors of loss of control eating in primary school children: A retrospective case-control study. Int J Eat Disord. 2012;45(6):751–8. 22431297 10.1002/eat.22018

[24] Bould H, Koupil I, Dalman C, DeStavola B, Lewis G, Magnusson C. Parental mental illness and eating disorders in offspring. Int J Eat Disord. 2015;48(4):383–91. 24965548 10.1002/eat.22325

[25] Martinson LE, Esposito-Smythers C, Blalock DV. The effects of parental mental health and social‐emotional coping on adolescent eating disorder attitudes and behaviors. J Adolesc. 2016;52(1):154–61. 27567519 10.1016/j.adolescence.2016.08.007 PMC5028292

[26] Figueiredo RADO, Simola-Ström S, Rounge TB, Viljakainen H, Eriksson JG, Roos E, et al. Cohort Profile: The Finnish Health in Teens (Fin-HIT) study: a population-based study. Int J Epidemiol. 2019;48(1):23–h24. 30212855 10.1093/ije/dyy189 PMC6380305

[27] Field AE, Taylor CB, Celio A, Colditz GA. Comparison of self-report to interview assessment of bulimic behaviors among preadolescent and adolescent girls and boys. Int J Eat Disord. 2004;35(1):86–92. 14705161 10.1002/eat.10220

[28] Figueiredo RADO, Simola-Ström S, Isomaa R, Weiderpass E. Body dissatisfaction and disordered eating symptoms in Finnish preadolescents. Eat Disord. 2019;27(1):34–51. 30040544 10.1080/10640266.2018.1499335

[29] Collins ME. Body Figure Perceptions and Preferences Among Preadolescent Children: International Journal of Eating Disorders. Int J Eat Disord. 1991;10(2):199–208.

[30] Rosenberg M. Society and the adolescent self-image. Princeton, USA: Princeton University Press. 1965.

[31] Maloney MJ, McGuire J, Daniels SR, Specker B. Dieting behavior and eating attitudes in children. Pediatrics. 1989;84(3):482–9. 2788865

[32] Lommi S, Viljakainen HT, Weiderpass E, de Oliveira Figueiredo RA. Children’s Eating Attitudes Test (ChEAT): a validation study in Finnish children. Eat Weight Disord. 2020;25(4):961–71. 31119587 10.1007/s40519-019-00712-w PMC7399682

[33] Beck AT, Beck RW. Screening depressed patients in family practice. A rapid technic. Postgrad Med. 1972;52(6):81–5. 4635613 10.1080/00325481.1972.11713319

[34] Beck AT, Rial WY, Rickels K. Short form of depression inventory: cross-validation. Psychol Rep. 1974;34(3):1184–6. 4424377

[35] Raitasalo R, Mood Questionnaire. Finnish Modification of the Short Form of the Beck Depression Inventory Measuring Depression Symptoms and Self-Esteem. [Mielialakysely. Suomen Oloihin Beckin Lyhyen Depressiokyselyn Pohjalta Kehitetty Masennusoireilun Ja Itsetunnon Kysely: Print Book in Finnish]. Helsinki: The Social Insurance Institution; 2007. (Studies in Social Security and Health; vol. 86).

[36] Bennett DS, Ambrosini PJ, Bianchi M, Barnett D, Metz C, Rabinovich H. Relationship of Beck Depression Inventory factors to depression among adolescents. J Affect Disord. 1997;45(3):127–34. 9298425 10.1016/s0165-0327(97)00045-1

[37] Sarkkola C, Rounge TB, Simola-Ström S, von Kraemer S, Roos E, Weiderpass E. Validity of home-measured height, weight and waist circumference among adolescents. Eur J Public Health. 2016;26(6):975–7. 27578829 10.1093/eurpub/ckw133

[38] Cole TJ, Lobstein T. Extended international (IOTF) body mass index cut-offs for thinness, overweight and obesity. Pediatr Obes. 2012;7(4):284–94. 22715120 10.1111/j.2047-6310.2012.00064.x

[39] Brechan I, Kvalem IL. Relationship between body dissatisfaction and disordered eating: Mediating role of self-esteem and depression. Eat Behav. 2015;17:49–58. 25574864 10.1016/j.eatbeh.2014.12.008

[40] Cruz-Sáez S, Pascual A, Wlodarczyk A, Echeburúa E. The effect of body dissatisfaction on disordered eating: The mediating role of self-esteem and negative affect in male and female adolescents. J Health Psychol. 2020;25(8):1098–108. 30101609 10.1177/1359105317748734

[41] March H. Global Self-Esteem: Its Relation to Specific Facets of Self-Concept and Their Importance. [cited 2024 Jun 20]. https://oce-ovid-com.libproxy.helsinki.fi/article/00005205-198612000-00016/PDF

[42] Ahrberg M, Trojca D, Nasrawi N, Vocks S. Body Image Disturbance in Binge Eating Disorder: A Review. Eur Eat Disorders Rev. 2011;19(5):375–81. 10.1002/erv.1100 21341345

[43] Chua JYX, Tam W, Shorey S. Research Review: Effectiveness of universal eating disorder prevention interventions in improving body image among children: a systematic review and meta-analysis. J Child Psychol Psychiatry. 2020;61(5):522–35. 31746023 10.1111/jcpp.13164

[44] Svantorp-Tveiten KME, Ivarsson A, Torstveit MK, Sundgot-Borgen C, Mathisen TF, Bratland-Sanda S, et al. The Healthy Body Image Intervention and Reduction in Eating Disorder Symptomatology and Muscle Building Supplement Use in High School Students: A Study of Mediating Factors. Front Psychol. 2022;13:803654. 35837620 10.3389/fpsyg.2022.803654 PMC9274278

[45] Stice E, Onipede ZA, Marti CN. A meta-analytic review of trials that tested whether eating disorder prevention programs prevent eating disorder onset. Clin Psychol Rev. 2021;87:102046. 34048952 10.1016/j.cpr.2021.102046

[46] He J, Cai Z, Fan X. Prevalence of binge and loss of control eating among children and adolescents with overweight and obesity: An exploratory meta-analysis. Int J Eat Disord. 2017;50(2):91–103. 28039879 10.1002/eat.22661

[47] Reas DL, Grilo CM. Timing and sequence of the onset of overweight, dieting, and binge eating in overweight patients with binge eating disorder. Int J Eat Disord. 2007;40(2):165–70. 17089414 10.1002/eat.20353

[48] Puhl RM, Wall MM, Chen C, Bryn Austin S, Eisenberg ME, Neumark-Sztainer D. Experiences of weight teasing in adolescence and weight-related outcomes in adulthood: A 15-year longitudinal study. Prev Med. 2017;100:173–9. 28450124 10.1016/j.ypmed.2017.04.023 PMC5852667

[49] Abdulkadir M, Herle M, De Stavola BL, Hübel C, Santos Ferreira DL, Loos RJF, et al. Polygenic Score for Body Mass Index Is Associated with Disordered Eating in a General Population Cohort. J Clin Med. 2020;9(4):1187. 32326247 10.3390/jcm9041187 PMC7231239

[50] Brun I, Russell-Mayhew S, Mudry T. Last Word: Ending the intergenerational transmission of body dissatisfaction and disordered eating: a call to investigate the mother-daughter relationship. Eat Disord. 2021;29(6):591–8. 32142392 10.1080/10640266.2020.1712635

[51] Armstrong B, Janicke DM. Differentiating the effects of maternal and peer encouragement to diet on child weight control attitudes and behaviors. Appetite. 2012;59(3):723–9. 22885728 10.1016/j.appet.2012.06.022

[52] Neumark-Sztainer D, Bauer KW, Friend S, Hannan PJ, Story M, Berge JM. Family weight talk and dieting: How much do they matter for body dissatisfaction and disordered eating behaviors in adolescent girls? J Adolesc Health. 2010;47(3):270–6. 20708566 10.1016/j.jadohealth.2010.02.001 PMC2921129

[53] Lommi S, Figueiredo RA, de Tuorila O, Viljakainen H. Frequent use of selected sugary products associates with thinness, but not overweight during preadolescence: a cross-sectional study. Br J Nutr. 2020;124(6):631–40. 32312332 10.1017/S0007114520001361 PMC7525105

[54] Rosenberg M. Society and the Adolescent Self-Image, Prevised edition. Middletown, CT, USA: Wesleyan University. 1989.

[55] Decaluwé V, Braet C. Prevalence of binge-eating disorder in obese children and adolescents seeking weight-loss treatment. Int J Obes Relat Metab Disord. 2003;27(3):404–9. 12629570 10.1038/sj.ijo.0802233

